# The turbocharged wide anterolateral thigh perforator flap to reconstruct massive soft tissue defects in traumatized lower extremities: A case series

**DOI:** 10.3389/fsurg.2022.991094

**Published:** 2022-10-25

**Authors:** Seong-Ho Jeong, Do-Yoon Koo, Kyung-Chul Moon, Eun-Sang Dhong, Seung-Kyu Han

**Affiliations:** Department of Plastic Surgery, Korea University Guro Hospital, Seoul, South Korea

**Keywords:** turbocharging procedure, anterolateral thigh perforator flap, extensive soft tissue defects, lower extremity trauma, branches of lateral circumflex femoral artery, limb salvage

## Abstract

**Background:**

Extensive traumatic soft tissue defects in the lower extremities typically require complete coverage of exposed bone because inadequate coverage, such as partial flap loss, may result in bony infection and ultimately lead to limb salvage failure. To achieve complete coverage of these defects, we used the wide anterolateral thigh perforator flap in which the turbocharging procedure augments the blood flow. Herein, we describe our turbocharging technique and discuss its effectiveness.

**Methods:**

From January 2014 to December 2020, the turbocharged wide ALTP free flaps were used to treat 13 patients with massive traumatic soft tissue defects in the lower extremities, ranging in size from 22 × 10 cm^2^ (220 cm^2^) to 21 × 17 cm^2^ (357 cm^2^) (mean, 270 cm^2^). All ALTP flaps were supplied by perforators from both the transverse branch of the lateral circumflex artery (TB-LCFA) and descending branch of the lateral circumflex artery (DB-LCFA) simultaneously. The turbocharging procedure by connecting the TB-LCFA to a side branch of the DB-LCFA was carried out in all these flaps. A retrospective review of medical records for each patient was performed.

**Results:**

The size of the transferred ALTP flap ranged from 23 × 12 cm^2^ (276 cm^2^) to 23 × 19 (437 cm^2^) (mean, 331 cm^2^). The total number of perforators included in the flaps was three on average. All ALTP flaps survived completely without partial necrosis. The postoperative course was uneventful except for two cases with minor complications, including hematoma and partial necrosis of the recipient's skin.

**Conclusion:**

Free transfer of the turbocharged wide ALTP flap can be a reliable and effective reconstructive method to obtain complete coverage of extensive traumatic soft tissue defects in the lower extremities and achieve successful limb salvage.

## Introduction

The lower extremity is one of the most frequently injured body parts in high-energy traumas, such as motor vehicle accidents and falls from height. Once high-energy traumatic injuries occur in the lower limbs, the injuries typically result in heavily contaminated open tibia fractures, which require repeated radical debridement. However, multiple debridements may create extensive soft tissue defects with exposed bone in some patients, and reconstructing these defects can be technically challenging, even for experienced surgeons.

In patients with severe open tibia fractures accompanied by extensive soft tissue defects, the final goal of treatment is to restore gait function by preserving the standard length and sensation of the affected extremity (1, 2). Achieving this goal requires a collaborative approach between orthopedic and plastic surgeons to simultaneously manage complex fractures and massive soft tissue defects. Generally, orthopedic surgeons debride all devitalized tissues and temporarily fixate fractured bones, and plastic surgeons attempt to reconstruct massive soft-tissue defects created by debridement.

Transfer of an extensive skin paddle is generally needed for soft tissue reconstruction to amply cover the defects and their surrounding areas that may be potentially damaged. Although surgeons can choose one of the various reconstructive options for this purpose (3), it is widely accepted that free tissue transfer employing microsurgical techniques is the ideal option for these massive bone and soft tissue defects in the lower extremities (4). However, microsurgical reconstruction using free flaps is technically demanding and has a long learning curve. Thus, a strategic approach with thorough preparation is mandatory to avoid limb salvage failures.

There have been several reports on free flap options for reconstructing extensive soft tissue defects in the lower extremities (3–9). Diverse reconstructive options using a single tissue or a combination of various body tissues have been introduced in these reports; however, the anterolateral thigh perforator (ALTP) flap is currently recognized as the most promising choice owing to its proven vascular reliability and versatility (10–14). The ALTP flap offers many benefits, including thin, malleable skin; useful pedicle vessels with sufficient length and caliber; and the ability to be harvested as various combinations of tissues, such as skin, fascia, and muscle (11, 15). Although the ALTP flap has been widely used to reconstruct massive soft tissue defects, the maximum size of the ALTP flap, free from the risk of partial flap necrosis, has not yet been clearly determined. However, some surgeons (15–18) have suggested that the upper limit of skin paddles that can be harvested based on a single perforator ranges from 240 to 630 cm^2^.

**Figure 1:**
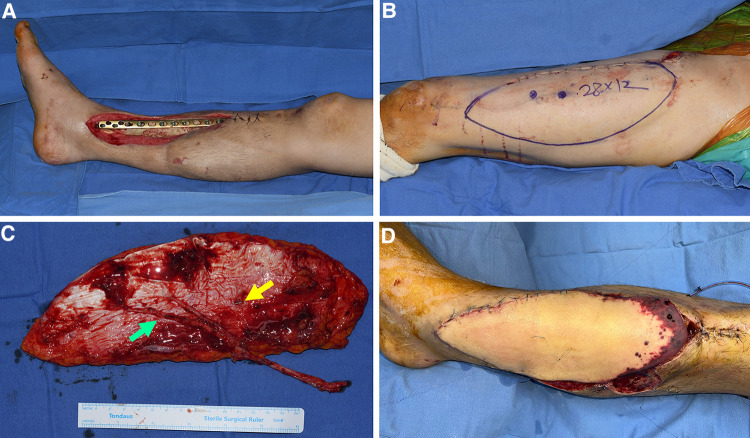
A case with partial necrosis of the transferred wide ALTP flap. (**A**) After radical debridement of necrotic bone and soft tissue, temporary bony fixation using a metal plate and antibiotics-loaded bone cement was performed on a 48-year-old male patient who sustained a traffic accident. (**B**) A wide ALTP flap (28 × 12 cm^2^) was harvested. (**C**) Although the flap was supplied by perforators from both TB-LCFA and DB-LCFA simultaneously, the flap was harvested, including only perforators from DB-LCFA after sacrificing a perforator from TB-LCFA (yellow arrow indicates a perforator from TB-LCFA; green arrow indicates DB-LCFA). (**D**) Venous congestion occurred 8 h after the surgery, leading to partial necrosis even after continuous medicinal leech therapy. ALTP, anterolateral thigh perforator; TB-LCFA, transverse branch of lateral circumflex femoral artery; DB-LCFA, descending branch of lateral circumflex femoral artery.

However, we encountered partial necrosis of some wide ALTP flaps despite limiting the flap size to within the previously suggested safe dimension, even in cases with multiple perforators (Figure 1). Case analysis revealed that these flaps initially included perforators from both the descending branch of the lateral circumflex femoral artery (DB-LCFA) and the transverse branch of the lateral circumflex femoral artery (TB-LCFA) before harvest. Nevertheless, the final elevated flap was supplied only by perforators from the DB-LCFA after severing the perforator from the TB-LCFA. We assumed that insufficient blood circulation following the sacrifice of the perforator from the TB-LCFA resulted in partial necrosis of some flaps.

Therefore, we have performed the wide ALTP flap transfer using a turbocharging procedure where the perforator from TB-LCFA was connected to the side branch of the DB-LCFA. This study retrospectively reviews our experiences in these turbocharged wide ALTP flap transfers. To our knowledge, there have been no organized studies except for some case reports on turbocharged ALTP flap transfer. This study aimed to describe the details of our turbocharging technique in the free transfer of a wide ALTP flap and demonstrate its effectiveness in reconstructing traumatic extensive soft tissue defects of lower extremities.

## Patients and methods

From January 2014 to December 2020, 13 patients (2 women, 11 men) who underwent reconstruction of traumatic lower extremity defects using free transfer of turbocharged wide ALTP flaps were identified and included in this study. In all these patients, the ALTP flaps were supplied by perforators originating from both TB-LCFA and DB-LCFA simultaneously, and the turbocharging procedure between TB-LCFA and DB-LCFA was performed after confirming the occurrence of insufficient blood circulation within the flap following temporary clamping of the perforators originating TB-LCFA.

**Figure 2:**
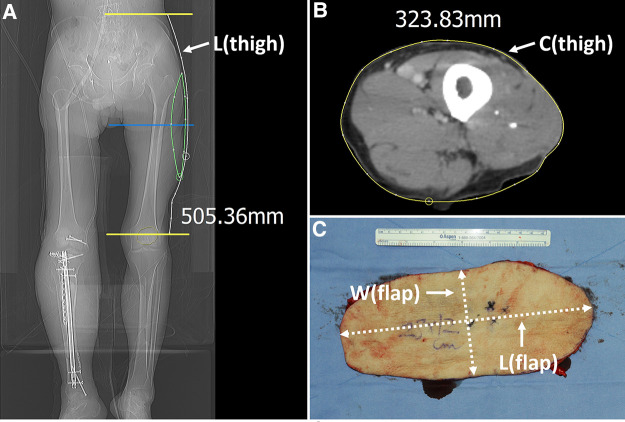
Measurement of the length of the thigh regions and the harvested flap to calculate length and width ratio. (**A**) The length between ASIS and patella center, which is named thigh length, was measured using a digital tool in the PACS. (**B**) The thigh circumference was measured using the curve measurement tool in PACS. (**C**) The major and minor axis length was measured from the harvested flap. ASIS, anterior superior iliac spine; PACS, picture archiving and communication system; L(thigh), length from ASIS to patella center; C(thigh), circumference of the thigh at the mid-level between ASIS and patella center; W(flap), width of the flap or length of the minor axis of the flap; L(flap), length of the major axis of the flap.

We performed a retrospective, single-center review of all medical records of these patients. Patients' information is summarized in Table 1. The patients' ages ranged from 19 to 73 years, with an average of 46 years. All the patients had Gustilo–Anderson type IIIB open fractures caused by road traffic or industrial accidents. Soft tissue defects developed following debridement, ranging from 22 × 10 cm^2^ (220 cm^2^) to 21 × 17 cm^2^ (357 cm^2^) (mean, 270 cm^2^). To objectively evaluate flap size, we calculate the ratio of the major axis length of the flap (Figure 2C) to the thigh length, measured from the ASIS to the patella center, using radiologic images (Figure 2A). We named this as length ratio. In addition, we also work out the width ratio, which implies the ratio of the flap width (minor axis length of the flap; Figure 2C) to the thigh circumference, measured using the curve measurement tool in PACS (Figure 2B). Eleven defects were located in the calf, and one in the foot. All surgical procedures were performed at the Department of Plastic Surgery, Korea University Guro Hospital, Seoul, Korea. The study followed the guidelines of the ethical committee of Korea University Guro Hospital in accordance with the ethical standards of the Helsinki Declaration of 1975.

**Table 1:** Summary of patients.

No	SEX/Age	Flap Feature			Perforator No			Anastomosis			Length ratio^c^	Width ratio^d^	Flap Outcome	Cx	Op Time	FU
		Site (Lower Leg)	Size (cm)	Donor	Dc	Tv	Turbo^a^	Recipient Artery	Vein^b^							
1	M/69	Foot, Heel, Rt	22 × 15	STSG	1	1	BeforeH	PTA	EtoE	2	0.45	0.42	Survival	None	7 h	3y
2	F/21	Lower, Ant, Rt	25 × 13	STSG	2	1	BeforeH	ATA	EtoE	2	0.52	0.35	Survival	None	10 h	4y1m
3	F/20	Lower, Ant, Rt	24 × 12	1′repair	2	2	BeforeH	ATA	EtoE	2	0.49	0.33	Survival	None	8 h	3y2m
4	M/70	Upper, Med, Rt	23 × 19	STSG	3	1	BeforeH	ATA	EtoS	1	0.44	0.48	Survival	None	8 h	2y3m
5	M/65	Whole Leg, Lt	25 × 14	STSG	1	1	AfterH	ATA	EtoS	2	0.49	0.42	Survival	None	12 h	1m
6	M/55	Middle, Med, Rt	23 × 12	STSG	1	1	AfterH	PTA	EtoS	1	0.46	0.32	Survival	None	9 h	5y8m
7	M/73	Whole Leg, Rt	28 × 12	STSG	2	1	BeforeH	ATA	EtoS	2	0.55	0.37	Survival	Skin Necrosis^e^	11 h	2y6m
8	M/42	Middle, Ant, Lt	23 × 12	STSG	2	1	BeforeH	ATA	EtoE	1	0.45	0.37	Survival	None	8 h	1y10m
9	M/23	Middle, Med, Rt	23 × 13	STSG	2	1	AfterH	ATA	EtoE	2	0.46	0.39	Survival	None	9 h	2y4m
10	M/33	Middle, Med, Lt	23 × 12	STSG	2	1	BeforeH	ATA	EtoS	2	0.47	0.39	Survival	None	7 h	3y6m
11	M/19	Upper, Ant, Rt	25 × 15	STSG	3	1	BeforeH	ATA	EtoE	2	0.49	0.40	Survival	None	8 h	5y1m
12	M/54	Upper, Ant, Rt	29 × 14	STSG	2	2	BeforeH	PTA	EtoS	2	0.56	0.43	Survival	Hematoma	12 h	1y9m
13	M/58	Lower, Ant, Lt	24 × 14	STSG	2	1	AfterH	ATA	EtoS	2	0.50	0.38	Survival	None	10 h	3y

No, number; Rt, right; Lt, left; Ant, anterior side; Med, medial side; Dc, Descending branch; Tv, Transverse branch; Turbo, Turbocharging; PTA, posterior tibial artery; ATA, anterior tibial artery; EtoE, end to end anastomosis; EtoS, end to side anastomosis; Cx, complications; PostOP, postoperative; BeforeH, before harvest of the flap at the donor site; AfterT, after harvest at the recipient site; FU, follow-up period; h, hour; y, year; m, month.

^a^ Timing and location of turbocharging.

^b^ Number of venous anastomosis.

^c^ Length ratio is defined as the ratio of the major axis length of the flap to the thigh length (measured from ASIS to patella center).

^d^ Width ratio is defined as the ratio of the flap width (minor axis length of the flap) to the thigh circumference.

^e^ Skin necrosis of recipient area.

### Surgical technique

All patients underwent preoperative computed tomography-assisted angiography and venography to assess the vascular condition of the recipient site and donor site of the ALTP flap. Preoperative mapping of the perforators in anterolateral thigh region was also performed using a hand-held Doppler. After drawing a line between the anterior superior iliac spine and the upper lateral border of the patella, we attempted to mark almost all perforators around this axis. Both orthopedic and plastic surgeons performed the surgical procedure simultaneously.

At the beginning of the surgery, with the help of orthopedic surgeons, a provisional template that reflects the presumptive size and shape of the soft tissue defect following debridement was made using a transparent film. Based on this template, we designed an ALTP flap on the contralateral thigh with its central portion matching the marking site of potentially reliable perforators. While orthopedic surgeons performed radical debridement and reduction of the fractures, we harvested the ALTP flap. First, an incision was made into the skin and fascia at the medial border of the flap design. We then tried to find most of the perforators from the DB-LCFA and TB-LCFA in the subfascial plane. The incision was extended proximally or distally when wide surgical exposure was required for vessel dissection. Once sizable perforators were identified, they were traced back to the main trunk of the DB-LCFA or TB-LCFA by meticulous dissection of the muscle fibers or the intermuscular septum. The DB-LCFA was dissected proximally to cope with pedicle length requirements adequately. In contrast, the TB-LCFA was dissected limitedly until its trunk was exposed. During the proximal dissection of the DB-LCFA, the side branches were just exposed without ligation and severance.

**Figure 3:**
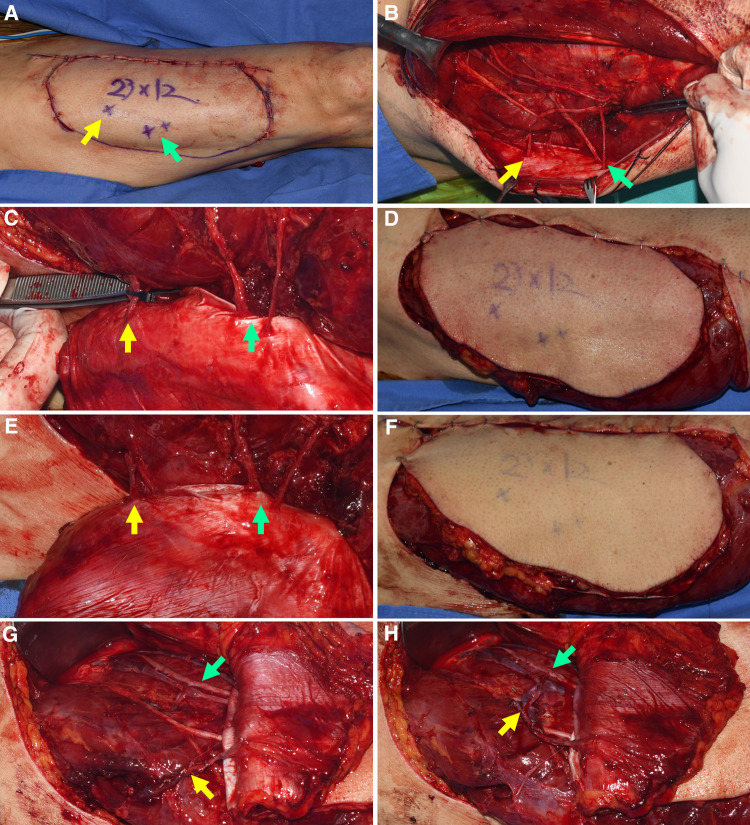
Determining whether the turbocharging procedure was required by temporary clamping the perforator from TB-LCFA (Each arrow indicates as follows: yellow indicates a perforator from TB-LCFA; green indicates DB-LCFA; white indicates side branches of DB-LCFA). (**A**) Based on the definite template, the flap design was adjusted to incorporate sizable perforators from both TB-LCFA and DB-LCFA. (**B**) One perforator from TB-LCFA and two perforators from DB-LCFA were isolated from muscles. (**C**) The blood flow in the perforator from TB-LCFA was blocked by temporary clamping using a microvascular single clamp. (**D**) Flap perfusion was assessed based on clinical examinations 30 min after clamping. The examinations revealed the dusky purple appearance of skin and brisk capillary refill, which indicated venous congestion. (**E**) The blood flow in the perforator from TB-LCFA was restored by removing the clamp. (**F**) The skin color and capillary refill time rapidly returned to normal. The results of the temporary clamping test implied that the turbocharging procedure where TB-LCFA was connected to the side branch of DB-LCFA was required. (**G,H**) The turbocharging procedure was performed before harvesting the flap. TB-LCFA, transverse branch of lateral circumflex femoral artery; DB-LCFA, descending branch of lateral circumflex femoral artery; ALTP, anterolateral thigh perforator.

**Figure 4:**
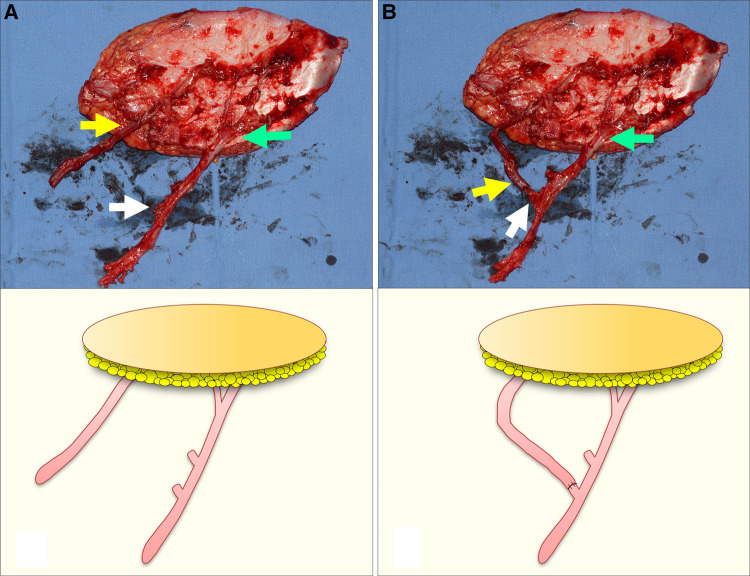
A simulation for demonstrating the turbocharging procedure in the wide ALTP flap supplied by perforators from both TB-LCFA and DB-LCFA (Each arrow indicates as follows: yellow indicates a perforator from TB-LCFA; green indicates DB-LCFA; white indicates side branches of DB-LCFA). (**A**) Before the procedure, the TB-LCFA of adequate length and the DB-LCFA with several potential candidate side branches were prepared. (**B**) The TB-LCFA was transposed to the selected side branches of the DB-LCFA, and the anastomosis between them was performed. ALTP, anterolateral thigh perforator; TB-LCFA, transverse branch of lateral circumflex femoral artery; DB-LCFA, descending branch of lateral circumflex femoral artery.

We then created a definite template by drawing on top of the final soft tissue defects following orthopedic debridement. Next, we adjusted the previous flap design to incorporate sizable perforators from the TB-LCFA and DB-LCFA by repeated application of this template on the lateral thigh. However, the medial border of the previous design was maintained. After confirming the design, an additional incision was made along the lateral border, and the flap was entirely elevated from the muscles. Before harvesting the flap, we determined whether the turbocharging procedure was required or not. At first, we clamped the perforator of TB-LCFA with a microvascular clamp (Figures 3A–D) and assessed the flap perfusion by physical examinations 30 min after clamping. Next, we released the clamp and re-assessed the flap perfusion (Figures 3E–H). When the results of this temporary clamping test revealed the necessity for the turbocharging procedure, we attempted to connect the TB-LCFA to a side branch of the DB-LCFA (Figure 4). Generally, we tried to perform microvascular anastomosis in one artery and two veins. However, in some cases, the side branch of DB-LCFA selected for anastomosis had only one available vein. Accordingly, anastomosis in one artery and one vein was carried out in these cases. (Table 1).

**Figure 5:**
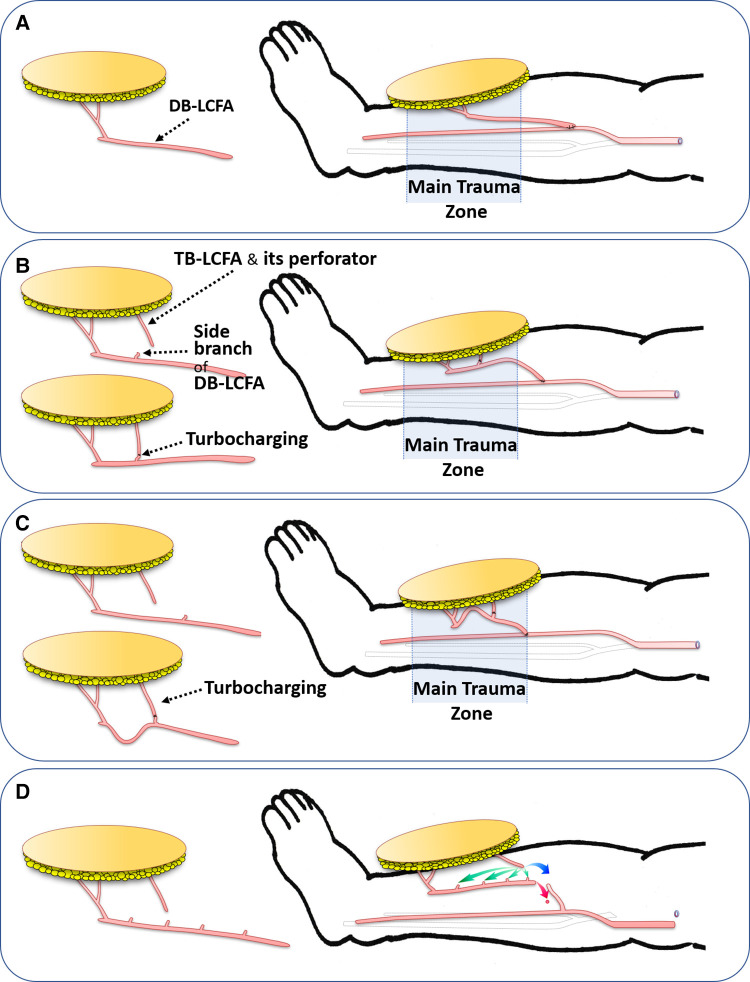
The effects of the turbocharging procedure on the main pedicle (DB-LCFA) length according to the timing of the procedure (Pre-or Post-harvest). (**A**) Without the turbocharging procedure, the transferred ALTP flap probably has great mobility of the main pedicle, enabling tension-free insetting of the flap. (**B**) With the turbocharging procedure, the length of the main pedicle possibly is shortened, and the mobility of the flap may be somewhat limited because of the fixation effect of the turbocharged vessel. (**C**) When the TB-LCFA is connected to the proximally located side branches of DB-LCFA, the main pedicle length may be critically shortened, precluding the main pedicle anastomosis from being performed proximal to the zone of injury. (**D**) In worrisome cases in which critical shortening of the main pedicle following the turbocharging procedure may occur, the ALTP flap is harvested and transferred without the turbocharging procedure. Next, the main flap pedicle is anastomosed to the recipient vessel in advance (red arrow). Then, the turbocharging procedure is attempted after appropriate preparation of the TB-LCFA and side branches of the DB-LCFA (green arrow). If it is impossible due to problems in the length or location of the vessels, vessel grafts or anastomosis between TB-LCFA and a side branch of the recipient's vessel (supercharging procedures; blue arrow) can be used to overcome those problems. DB-LCFA, descending branch of lateral circumflex femoral artery; ALTP, anterolateral thigh perforator; TB-LCFA, transverse branch of lateral circumflex femoral artery.

The turbocharging procedure can augment the blood supply to the flap by adding a new supplying vessel to the flap pedicle. However, adding a vessel to the pedicle may shorten the pedicle length and lead to difficulty in pedicle anastomosis to recipient vessels (Figures 5A–C). Accordingly, before performing the turbocharging procedure, we tried to predetermine the optimal length and location of the newly created vessel by the procedure to avoid problematic shortages of the main pedicle following the turbocharging procedure. Thus, the recipient vessels, such as the anterior or posterior tibial vessels, were dissected before harvesting the flap. We then estimated the required length of the flap pedicle and its future course from the skin paddle of the flap to the recipient vessel. Based on these estimations, we determined the proper length of the TB-LCFA and selected the most appropriate side branches of the DB-LCFA for turbocharging procedure (Figures 5B,C).

Furthermore, according to the findings at the donor and recipient sites, we decided the timing of the turbocharging procedure. When the critical shortage of the main pedicle seemed to be avoidable, we performed a turbocharging procedure before the complete harvest of the ALTP flap. Generally, the turbocharging procedure could be carried out more comfortably and straightforwardly at the donor site before the flap harvest. However, when a significant reduction in the main pedicle length seemed inevitable, or the main pedicle length after turbocharging was unpredictable, we decided to carry it out after transferring the flap to the recipient site.

In the former case (when turbocharging was done before harvesting the ALTP flap), additional pedicle dissection was conducted until the predetermined length of TB-LCFA was achieved, and most of the side branches of DB-LCFA, except the preselected ones, were clipped and cut. After full skeletonization of vessels, the TB-LCFA was severed at the predetermined level, and it was transposed and anastomosed to the preselected side branch of the DB-LCFA. The ALTP flap was harvested by dividing the main pedicle at the required length and then transferred to the recipient site. The main pedicle were anastomosed to the prepared recipient vessels in the predetermined healthy area.

In the latter case (where turbocharging was done after the flap transfer), the TB-LCFA was dissected as proximally as possible, clipped and cut. In addition, the most sizable side branches of DB-LCFA were dissected approximately 1–2 cm in length, clipped, and cut. The ALTP flap was harvested by severing the main pedicle at the most proximal level and then transferred to the recipient site. The main pedicle were anastomosed to the recipient vessels at the most distal portion of the healthy area. The TB-LCFA was then transposed to the side branches of the DB-LCFA and anastomosed to the most appropriate side branch of the DB-LCFA, enabling a tension-free anastomosis (Figure 5D).

After the flap was located to maximally cover the soft tissue defects, the flap skin was sutured to the adjacent recipient skin or muscle. In 12 cases, the donor sites were closed with a split-thickness skin graft, whereas the other was closed primarily. Postoperatively, the flap was closely monitored hourly for color, turgor, and capillary refill. Arterial pulsation was also assessed hourly using a hand-held Doppler.

## Results

The size of the harvested ALTP flap ranged from 23 × 12 cm^2^ (276 cm^2^) to 23 × 19 (437 cm^2^) (mean, 331 cm^2^). The mean major axis length of the flap was 24.4 cm (22–29 cm), and the mean minor axis length (flap width) was 13.6 cm (12–19 cm). The mean thigh length was 50.1 cm (48–53 cm), and the mean thigh circumference was 34.9 (31–39 cm). The mean length ratio was 0.49 (0.44–0.56), and the mean width ratio was 0.39 (0.32–0.48).The number of perforators from the DB-LCFA included in the ALTP flap ranged from one to three, with two being the most common (62%). Conversely, only one perforator from the TB-LCFA was included in most cases, except in two cases with two perforators. The total number of perforators included ranged from two to four (mean, three). In nine cases, the turbocharging procedure was performed at the donor site before harvesting. In contrast, it was executed at the recipient site after harvesting, in four cases. The anterior tibial artery and its concomitant veins were used as recipient vessels in 10 cases, and the posterior tibial artery and its concomitant veins were used in three cases. The arterial anastomosis was performed in an end-to-end manner in six patients and in an end-to-side manner in seven patients. The end-to-end arterial anastomosis was performed when a disrupted artery could be used as the recipient artery. One or two venous anastomoses were performed in an end-to-end manner in all patients.

All turbocharged multiple perforator-based ALTP flaps were successfully transferred and survived without severe complications. However, partial necrosis of the recipient's skin adjacent to the transferred flap occurred because of skin undermining in one patient and was ultimately treated with debridement and dressing. Another patient developed a postoperative hematoma, which was resolved without any adverse effect on the flap. All donor sites healed perfectly, without requiring additional procedures.The mean total operation time was 9.4 h (range: 6.7–12.4 h), and the mean operation time of the turbocharging procedure was 45 min (range: 32–66 min). The duration of the postoperative follow-up period ranged from 1 to 68 months (mean, 35 months). All patients were able to ambulate with or without the assistance of crutches at the last follow-up, except for one patient who died owing to a systemic illness, irrelevant to the flap surgery, one month postoperatively.

## Case reports

### Case 1 (No. 7)

**Figure 6:**
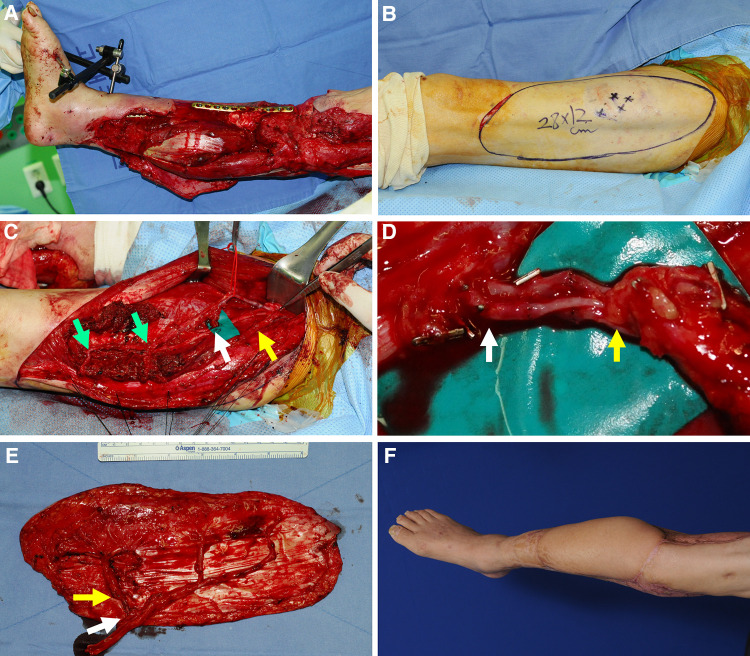
A clinical case showing reconstruction with a wide ALTP flap where the turbocharging procedure was performed before harvesting the flap. (Each arrow indicates as follows: yellow indicates a perforator from TB-LCFA; green indicates DB-LCFA; white indicates side branches of DB-LCFA). (**A**) A 73-year-old man presented with extensive soft tissue defects on the right lower leg caused by a traffic accident. (**B**) A wide ALTP flap (28 × 12 cm^2^) was elevated to cover exposed bone and plate after radical debridement and temporary bony fixation. (**C**) The ALTP flap included perforators from both DB-LCFA and TB-LCFA, and all vessels related to the turbocharging procedure were isolated and prepared. (**D**) The anastomosis was executed. (**E**) The harvested turbocharged wide ALTP flap (**F**) 16 months postoperatively. ALTP, anterolateral thigh perforator; TB-LCFA, transverse branch of lateral circumflex femoral artery; DB-LCFA, descending branch of lateral circumflex femoral artery.

A 73-year-old man was involved in a traffic accident and sustained substantial soft tissue injuries in the right lower leg (Figure 6A). Serial debridement to remove the devitalized skin and muscle resulted in extensive soft tissue defects. The necrotic bone was also removed and fixed with a plate by orthopedic surgeons after replacing the bone gap with antibiotic-loaded bone cement. A wide ALTP flap (28 × 12 cm^2^) was designed on the contralateral thigh for all exposed bones and plate coverage (Figure 6B). One perforator from the TB-LCFA and two perforators from the DB-LCFA were found during flap elevation. The turbocharging procedure was performed between these two types of perforators before flap harvesting because it was speculated that the main pedicle length would not be shortened after turbocharging (Figures 6C,D). Consequently, successful reconstruction was achieved with this turbocharged wide ALTP flap anastomosed to the anterior tibial artery in an end-to-end manner (Figures 6E,F). Additionally, the residual wound around the transferred flap and the donor site was covered with a split-thickness skin graft. The postoperative course was uneventful, without any severe complications. The patient could walk independently with a mild gait disturbance caused by stiffness of the ankle and knee joint at 30 months postoperatively (last follow-up).

### Case 2 (No. 5)

**Figure 7:**
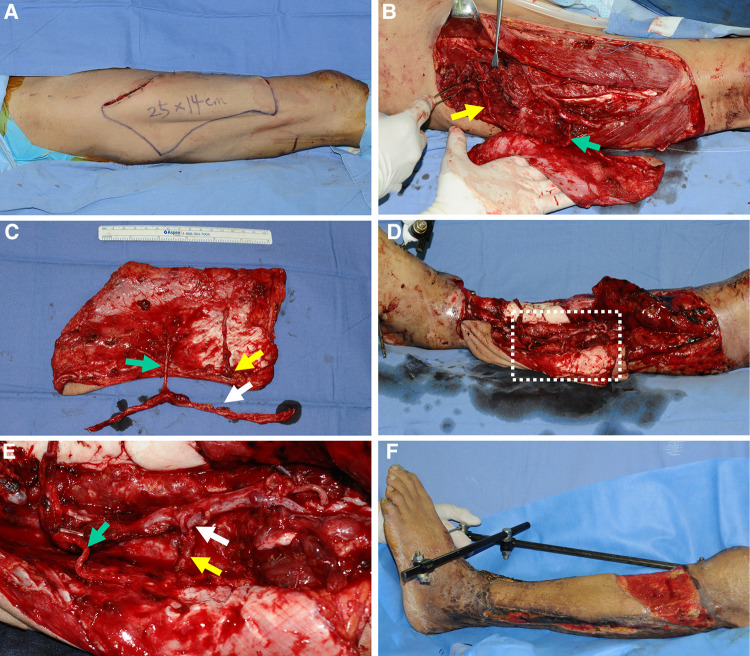
A clinical case demonstrating reconstruction with a wide ALTP flap where the turbocharging procedure was performed after harvesting the flap. (Each arrow indicates as follows: yellow indicates a perforator from TB-LCFA; green indicates DB-LCFA; white indicates side branches of DB-LCFA). (**A**) To reconstruct a massive soft tissue defect in the left lower leg, a wide ALTP flap (25 × 14 cm^2^) was designed on the contralateral thigh. (**B**) Perforators from both TB-LCFA and DB-LCFA supplied the ALTP flap. (**C**) The flap was harvested and transferred to the recipient site before the turbocharging procedure because its effects on the main pedicle anastomosis could not be assessed due to the ambiguous vessel anatomy in the destructed recipient wounds. (**D**) The main pedicle of the transferred ALTP flap was anastomosed to the recipient vessels, and the turbocharging procedure was performed. (**E**) An enlarged photograph of the part of the white dotted box in (**D**) shows the turbocharging procedure between TB-LCFA and a side branch of DB-LCFA (**E**) 23 days postoperatively. ALTP, anterolateral thigh perforator; TB-LCFA, transverse branch of lateral circumflex femoral artery; DB-LCFA, descending branch of lateral circumflex femoral artery.

A 65-year-old man sustained a severe crushing injury to his left lower leg while working at a manufacturing factory. Following radical debridement, massive soft tissue defects were observed. Therefore, we decided to achieve soft tissue coverage using an extended ALTP free flap (25 × 14 cm^2^) and designed the flap on the contralateral thigh based on a template reflecting the size and shape of the defect (Figure 7A). During the harvest of the ALTP flap, we found one perforator from the DB-LCFA and the other from the TB-LCFA. A turbocharging procedure was required to obtain stable blood circulation in the flap. After obtaining information on the future course, we estimated the length of the ALTP flap pedicle through recipient vessel dissection. We then attempted to postulate the optimal length of the TB-LCFA and the best side branch of the DB-LCFA for the turbocharging procedure; however, this was too difficult to confirm because the complexity of the recipient's wound hindered exact evaluation. Therefore, we planned to perform a turbocharging procedure at the recipient site. The designed flap was harvested with long TB-LCFA and DB-LCFA with multiple side branches (Figures 7B,C) and transferred to the recipient site. The DB-LCFA of the flap was connected to the anterior tibial vessels in advance. We then found a side branch of the DB-LCFA that could be anastomosed to the TB-LCFA without tension. Therefore, the turbocharging procedure was successfully executed (Figures 7D,E), and adequate coverage of soft tissue defects was obtained. The flap survived without any postoperative complication (Figure 7F). However, the patient died one month after the surgery because of sepsis caused by aspiration pneumonia (not related to reconstructive surgery).

### Case 3 (No. 11)

**Figure 8:**
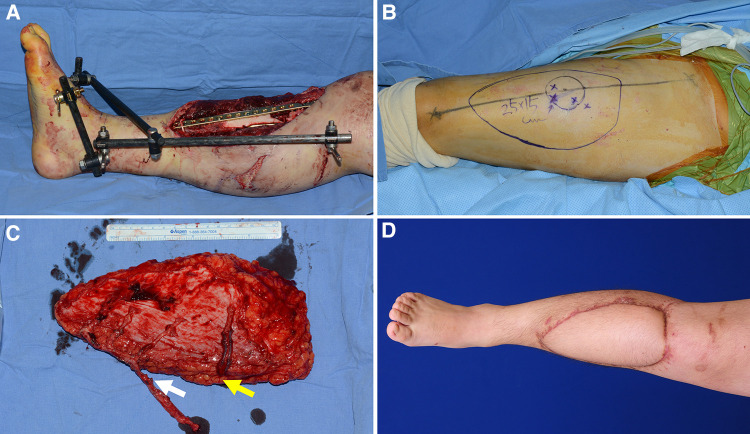
A clinical case presenting reconstruction with a wide ALTP flap where the turbocharging procedure was performed after harvesting the flap. (Each arrow indicates as follows: yellow indicates a perforator from TB-LCFA; white indicates side branches of DB-LCFA) (**A**) An 18-year-old man sustained severe bone and soft tissue injuries in the right lower leg from a motorcycle accident. (**B**) A wide ALTP flap (25 × 15 cm^2^) was designed. (**C**) The flap was harvested without the turbocharging procedure because the main pedicle was possibly expected to be critically shortened by adding a turbocharged supplying vessel because the flap already had three perforators from DB-LCFA. (**D**) A photograph was taken 21 months after reconstruction using a wide turbocharged ALTP flap.

A 19-year-old man was involved in a motorcycle accident, resulting in soft tissue defects in his right lower leg. Orthopedic surgeons performed radical debridement of necrotic soft tissue and bone. Soft tissue reconstruction was essential to cover the resulting massive defect and salvage the limb (Figure 8A). First, a wide ALTP flap (25 × 15 cm^2^), supplied by three perforators from the DB-LCFA and one perforator from the TB-LCFA, was elevated from the contralateral thigh (Figures 8B,C). The turbocharging procedure was performed after the flap was transferred to the recipient site. The addition of one more perforator to the main pedicle, which already had three perforators, seemed to significantly restrict its mobility (Figure 8C). Therefore, after connecting the main pedicle to the anterior tibial vessels, we selected the appropriate side branch of the DB-LCFA and performed an anastomosis between the selected branch and TB-LCFA. The flap completely survived (Figure 8D) and the patient was able to ambulate naturally without assistance at the last follow-up.

## Discussion

In patients with extensive soft tissue defects and bony exposure resulting from severe traumatic lower extremity injuries, the primary goal of reconstruction should be the complete coverage of exposed bone with a well-vascularized soft tissue. It is because incomplete coverage may incur bony infection and lead to limb salvage failure (19). Free tissue transfer of cutaneous tissues or muscles is ideal for achieving this goal regarding size and vascularity (3, 20). However, ensuring the complete survival of the free flap without partial necrosis is technically demanding. Thus, various methods using diverse tissues, either alone or in combination, have been used to reconstruct massive lower extremity defects.

Traditionally, the latissimus dorsi (LD) muscle flap has been considered a primary reconstructive option to meet this requirement because it can have extensive flap dimensions, strong resistance to infection, good accessibility, and well-known anatomy (21). However, it has some drawbacks, such as relatively short pedicle length, limited flap dimension not enough to cover extensive defects, and vulnerability to ischemic damage caused by vascular problems. In addition, recently, some researchers reported the harvest of LD muscle flaps could result in the functional limitation of the shoulder joint (22, 23). Therefore, with the advent of perforator flaps as a working horse in the field of microsurgical reconstruction, the LD muscle flap has been gradually replaced with the thoracodorsal artery perforator (TDAP) flap.

Usually, a conventional single free tissue transfer tends to be too small to adequately reconstruct massive lower extremity wounds (9). Therefore, some surgeons have advocated using modified single tissue flaps, such as the kiss deep inferior epigastric artery perforator (DIEP) flap (7) and bipedicled DIEP flap (8). On the contrary, other surgeons prefer to use the combined flap. These flaps are typically harvested from the back, for example, combined latissimus dorsi (LD)-serratus anterior flap or conjoined LD-parascapular flap (5) Sometimes, from the thigh, an anterolateral thigh (ALT) chimeric flap including skin paddle and muscle simultaneously (13) and ALT flow-through type flap connected by LD muscle (6) have also been used. Although these flaps can be a viable option to cover extensive lower limb defects, they have not been regarded as the gold standard method because procedures are technically demanding and usually accompanied by significant donor site morbidity.

Therefore, as an alternative, some surgeons have attempted to use a single wide ALTP free flap to cover extensive lower extremity defects (15). The ALTP flap is a versatile flap with a long pedicle and various-sized skin paddles. It is generally accepted that the ATP flap could be transferred with a single centrally located sizable perforator even though the skin paddle is extensive and formed into a particular shape by folding and suturing (24) or thinning procedure (25). Regarding soft tissue reconstruction in massively injured traumatic lower limbs, some authors achieved successful reconstructive results with extended ALTP flaps and have reported its reliability. According to these reports, the upper limit of flap dimensions ranged from 240 to 630 cm^2^. However, the maximum dimension of the ALTP flap that can be transferred without the risk of partial necrosis has not been confirmed yet because there have been no controlled studies on a large volume of cases (15, 26). In addition, the maximum size of a safely transferrable ALTP flap is likely to be different for each patient because of the difference in the thigh length and circumference. In this study, to evaluate the flap size objectively, we investigate the length and width ratio of the harvested ALTP flap in the thigh region. Although we could not collect meaningful data due to the small sample size, we found that it could be potentially helpful to decide on the optimal flap size individually for each patient. Therefore, in the future study, we are planning to measure the length and width ratio of the ALTP flap prospectively to determine the maximum length and width ratio of the safely transferrable ALTP flap.

Saint-Cyr et al. examined the mechanisms of blood supply to the ALTP flap through imaging studies on the vascularity of cadaveric flap samples. They showed that arterial flow through a perforator introduced into one vascular territory, called a perforasome, could expand to another through suprafascial linking vessels and by recurrent flow through the subdermal plexus (26–28). Furthermore, they suggested that wide perforator flaps, such as the extended ALTP flap, could be harvested *via* arterial linking vessels. According to their explanation, ligation of the side branches of a source artery could increase the blood flow within the selected perforator, resulting in dilation of the perforator itself and interperforator linking vessels. In other words, adequate blood flow to the entire flap can be naturally achieved by preparing the main pedicle. They also presented successful clinical cases that were reconstructed with an extended ALTP flap supplied by a single perforator with extensive skin paddles ranging 250 to 630 cm^2^ (26, 29).

However, we encountered cases in which partial necrosis caused by venous congestion occurred in the proximal region after the free transfer of wide ALTP flaps. These flaps initially had dual blood supply through perforators from the DB-LCFA and TB-LCFA. However, to facilitate flap harvest, TB-LCFA and its perforators were sacrificed, and the final transferred flap included only perforators from DB-LCFA. Consequently, contrary to our expectations, partial flap necrosis occurred in the proximal portion mainly supplied from TB-LCFA despite containing multiple sizable perforators from DB-LCFA in the flaps. In these cases, to manage partial necrosis, we had to transfer a secondary free flap at the risk of failure and carry out wound management for a long time, even though it could increase the chance of bony infection in the long term. Therefore, we were not sure that all ALTP flaps with extensive skin paddles could be transferred completely based on a single perforator or only perforators from DB-LCFA after sacrificing ones from TB-LCFA.

Anatomical studies have demonstrated the diverse branching patterns of the LCFA. Although the names of each branch are confusing because of their frequent change (30), the LCFA is traditionally known to have three branches: an ascending branch to the tensor fascia lata muscle, a transverse branch to the proximal portion of the vastus lateralis, and a descending branch to the distal portion of the vastus lateralis. Wong et al. described a new oblique branch running between the transverse and descending branches. Based on the intraoperative anatomical findings during flap elevation, they explained that the LCFA has three branches, including transverse, oblique, and descending branches (without the ascending branch). This branch is usually found lateral to the descending branch in the proximal thigh. It branches out of the musculocutaneous perforators through the vastus lateralis or septocutaneous perforators to the intermuscular septum between the rectus femoris and vastus lateralis. The clinical presentations of TB-LCFA in this study were similar to those of the oblique branch. Therefore, the perforators from the TB-LCFA seem to be the same as those from the oblique branch of the LCFA (31).

Taylor et al. (32–34) defined a specific cutaneous area supplied by a perforator as a “cutaneous perforator angiosome territory.” They performed cadaver injection studies of cutaneous perforators and showed that cutaneous perforator angiosome territories were interconnected *via* “choke arteries” and “valveless oscillating veins.” According to their studies, the choke arteries ordinarily remain constricted but can be dilated by increased blood flow, and the oscillating veins maintain the equilibrium of venous flow and pressure between the neighboring valved venous areas. Furthermore, they also demonstrated that when a flap was harvested based on a single cutaneous perforator, it safely included one neighboring cutaneous perforator angiosome territory in all directions through vascular connection *via* choke arteries and oscillating veins. However, they reported that when the flap had two neighboring territories connected in series, necrosis occurred around the second connected territory in 80 percent of cases in animal experiments.

These results imply that necrosis of a cutaneous perforator angiosome territory can occur after sacrificing its supplying perforator if the territory is located far from another territory which still supplied by its intact perforators. Accordingly, the leading cause of our partial flap necrosis cases may be that the distance between the proximal flap portion and the perforators from DB-LCFA was too far for the blood circulation to be maintained after sacrificing the perforators from TB-LCFA. However, determining the proximal limitation of a wide ALTP flap within a safe distance from the perforators of DB-LCFA is technically impossible due to anatomical variations and the lack of evaluation methods. Thus, preoperative flap size or design adjustment to avoid proximal flap necrosis is also impossible.

As the size of the ALTP flap increases, the number of perforators included in the flap tends to increase. Previous anatomical studies revealed that most of these perforators originated from the DB-LCFA and a relatively small number originated from the TB-LCFA (30, 35). However, not all ALTP flaps with wide skin paddles are supplied by perforators from the DB-LCFA and TB-LCFA simultaneously; some flaps could be supplied by both, while others by only one, because of the anatomical variation. In the wide ALTP flaps supplied by perforators from only one branch of the LCFA, insufficient blood circulation would probably not occur because the flap has a single angiosome consisting of several perforasomes connected by linking or choke arteries and oscillating veins. However, when the wide ALTP flaps, initially supplied by perforators from dual branches, are elevated after severing TB-LCFA, the farthest region of the flaps (supplied by the remaining perforators from DB-LCFA) may suffer from inadequate blood circulation because the flap initially had two angiosomes. Specifically, if the blood flow through the perforators from DB-LCFA is too weak to dilate linking vessels, such as the choke artery and oscillating veins, or if two angiosomes are in the vicinity of the connection through these linking vessels, the proximal portion of the angiosome supplied by TB-LCFA might be in danger of ischemic damage or venous congestion. Therefore, to enhance the blood circulation of the whole flap, we decided to perform a turbocharging procedure between TB-LCFA and DB-LCFA if they initially supplied wide ALTP flaps.

We elevated the flap with complete isolation of sizable perforators from both branches, disconnected both branches from LCFA, and performed the turbocharging procedure by connecting the severed TB-LCFA to a side branch of DB-LCFA. As a result, complete coverage of the defects without partial necrosis was achieved. Accordingly, we believe that the turbocharging procedure should be considered primarily in every case with available vascular anatomy to minimize the risk of partial necrosis, which is a critical problem leading to salvage failure in patients with extensive traumatic lower extremity injuries.

Supercharging and turbocharging are surgical techniques for enhancing blood flow to the flaps. Supercharging connects one of the supply vessels of a flap to the vessels outside the flap. In contrast, turbocharging involves interconnection of supplying vessels of a flap. There have been some reports on lower extremity reconstruction using a supercharged distally based ALTP rotation flap (24, 36). However, the supercharging technique is rarely used for free tissue transfer in traumatic lower extremity reconstruction because of the limited availability of healthy external vessels at the recipient's site owing to trauma. The turbocharging technique has been adopted during the harvest of deep inferior epigastric artery perforator flaps (37, 38). However, ALTP flaps are rarely harvested using this method because it is widely accepted that ALTP flaps with extensive skin paddles can be harvested safely, even based on a single perforator. In addition, the correlation between the number and size of the perforator vessels and ALTP flap survival has not been confirmed (29, 39). Nevertheless, to avoid partial flap loss, some surgeons recommend using the turbocharging technique when a wide ALTP flap should be harvested because they have experienced peripheral flap necrosis or are worried about its occurrence (40, 41). Although these reports do not have adequate scientific evidence due to the small number of patients, we agree with their opinions on the possibility of partial necrosis following wide free ALTP flap transfer and the potential usefulness of turbocharging procedure in those situations. Herein, we also suggest that the turbocharging procedure between TB-LCFA and DB-LCFA in the ALTP flaps, initially supplied by both, is required to secure a successful flap transfer based on our own experiences. Although this procedure imposes additional physical stress on surgeons and makes the surgery much more complex by affecting the course and length of the pedicle, we believe it could reward operators adequately with short-term assurance and long-term stability.

In patients enrolled in this study, a final decision on whether to implement the turbocharging procedure had been made after confirming insufficient blood circulation in a wide ALTP flap with the perforator clamping test. In particular, the test revealed venous congestion of the flap in all patients. This finding may denote that inadequate venous drainage is the main problem after sacrificing perforators originating from TB-LCFA. Saint-Cyr et al. (26–28, 29), based on imaging studies on the vascularity of cadaveric flap samples, demonstrated that venous flow in the anterior thigh region drained mainly through the superficial venous network on the subdermal plexus to the superficial veins, and minorly through the concomitant veins of perforators to the deep veins. They also showed that the venous flow would drain mainly through concomitant veins of the perforators after the elevation of the ALTP flaps because they usually do not include superficial veins. These results indicate that venous flow, normally drained through both superficial and deep venous systems, concentrates on the deep venous system, that is, concomitant veins of the perforators, which have relatively weak drainage functions. Therefore, the role of perforators seems to become more prominent in venous drainage of ALTP flaps. In this regard, the absence of perforators from TB-LCFA in wide ALTP flaps is more likely to result in venous congestion than arterial insufficiency. Our results from the perforator clamping tests support this presumption.

The turbocharging procedure was performed before or after harvesting the ALTP flap. When it is done before the flap harvest, anastomosis can be carried out more quickly because the required vessels are already designated. However, surgeons have less room to adjust the anastomotic point and flap location at the recipient site because adding a new supplying vessel to the pedicle could limit its mobility. Conversely, when turbocharging is done after the flap harvest, anastomosis can be time-consuming because the required vessels should be selected and prepared. However, tension-free anastomosis of the pedicle and flap insetting was possible because the location of the pedicle and skin paddle could be adjusted appropriately at the recipient site before the turbocharging procedure.

Owing to the small number of cases and the absence of a control group, our study has limitations in assessing the actual benefit of the turbocharging procedure between TB-LCFA and DB-LCFA in wide ALTP flap transfer. Therefore, we plan to conduct further studies to determine the necessity of the above turbocharging procedure and the critical size of the ALTP flap, in which this turbocharging procedure is mandatory. Concretely, we will analyze the occurrence of circulatory problems through conventional flap monitoring and indocyanine green perfusion tests after clamping the TB-LCFA in various ALTP flaps supplied by perforators from both TB-LCFA and DB-LCFA in a prospective manner.

## Conclusion

During the harvest of wide ALTP flaps supplied by perforators from both TB-LCFA and DB-LCFA, the turbocharging procedure, in which TB-LCFA was connected to a side branch of DB-LCFA, was performed to augment the blood supply to the whole flap and minimize partial flap loss. Using these turbocharged wide ALTP flaps, complete coverage of traumatic massive soft tissue defects in lower extremities was attained in all patients enrolled in this study. Therefore, we believe that the turbocharged wide ALTP flap could be a reliable and effective new option for obtaining complete coverage of extensive soft tissue defects in traumatized lower extremities and achieving limb salvage.

## Data Availability

The original contributions presented in the study are included in the article/Supplementary Material, further inquiries can be directed to the corresponding author/s.
